# Plant Intelligence from a Comparative Psychology Perspective

**DOI:** 10.3390/biology12060819

**Published:** 2023-06-05

**Authors:** Umberto Castiello

**Affiliations:** Dipartimento di Psicologia Generale, Università di Padova, 35121 Padova, Italy; umberto.castiello@unipd.it

After being subjected to years of debates regarding the possibility that plants possess some form of intelligence, many admit to needing to close their eyes and to breathe mindfully when having to listen to the same arguments yet again. On the one hand, there are some scientists from a wide range of disciplines including plant physiology, philosophy and psychology who argue that the complexity of plant responses to an ever-changing environment is indicative of intelligent behavior [1,2,3,4]. On the other hand, equally prestigious experts continue to insist that plant behavior does not meet the necessary conditions to be defined as intelligent [5,6,7]. Finally, there are groups of plant biologists, botanists, etc., who morph into philosophers and psychologists (and the latter groups who morph into the former ones) and contribute to the discussion with facile observations. Such a coalescence of disciplines might have been a good thing for plant science and science in general if it were not for the charged atmosphere in which the debate plunges forward.

So, getting to the topic of this editorial: Are plants intelligent? Yes, of course they are. Can anyone reasonably question the complexity of plant responses to an ever-changing environment? As has recently been pointed out, plant responsiveness emerges at all levels of plant research from genetic to phenotypic [8]. Doubting plant intelligence would mean challenging the notion that intelligence is a multifaceted concept, also defined as a “universal biological phenomenon promoting individual fitness and required for effective organism environment interactions” [9].

Is plant and animal intelligence the same? No, of course not. Plant intelligence is not, for example, neurocentric. Additionally, animal intelligence is unquestionably different from plant intelligence as each has evolved in the face of its own specific ecological needs. What is more, animal intelligence relies on strategies that can efficaciously deal with fast movements. Plant intelligence, instead, depends on mechanisms that can successfully promote sessile organisms whose movements can be rapid (i.e., the snap trap mechanisms of carnivorous plants or leaflet folding mechanisms of some Fabaceae species). For the most part, however, their movements are so slow as to be imperceptible to the naked eye. Generally speaking, we think of plants as being relatively immobile, permanently restricted to a substratum or base. This is a critical point as it may be the one major reason why we perceive plants as bereft of intelligence. To explain, if someone were to ask you to name an intelligent organism, I doubt you would say an orchid. In fact, we are used to assigning intelligence to organisms that move about on their own; in other words, we tend to assume that organisms that move in certain spatio-temporal conditions have intentions and thus possess some form of intelligence [10,11]. Although by no means passive, most plants are (apparently) incapable of moving from one place to another; therefore, they seem incapable of intelligent behavior. Using this criterion, intelligence and movement (behavior) seem to be inextricably connected. Instead, equating the ability to move to intelligence is both anthropomorphic as well inappropriate as plants do indeed move (even a lot) in a variety of ways [12].

Following this line of reasoning, would it change our minds about plant intelligence if we could see their movement? Would that be enough to convince us that plants are intelligent?

There is a way for us to see and observe the movement of plants: time-lapse videos that speed up plant movements, bringing them to a ‘human’ perception level. This methodological approach does not serve to make plant movement resemble (artificially) the movement of animals, but rather to make their intelligent behavior more obvious. We can use this strategy to observe the real nature of plants intelligence based on their behavior, and this could be the stepping stone to a comparative approach [4]. Just as we can watch videos of animals in slow motion so we can observe ‘hidden’ details permitting us to make comparisons across species, we can view time-lapse videos of plants exposing meaningful variations, permitting us to make comparisons across species.

I ask the reader to consider: why should plants be excluded from the comparative intelligence debate? Does the fact that plants are brainless preclude them from being intelligent? The idea that plant behavior might not be intelligent because plants do not have brains implies, from a “neurocentric” perspective, that brains or central nervous systems are intelligent. However, from a realistic biological perspective, brains per se are not “intelligent”. In and throughout the totality of their body organization, plants show intelligent behavior by using appropriate strategies to adapt to an ever-changing environment [3]. Another subtle error commonly made by sceptics of plant intelligence is assuming that behavior is only an external manifestation of something “internal” (in this case, intelligence). Intelligent behavior can denote intelligence; in the case of plants, it is the outcome of multiple processes that could be called intelligence in action [3,13].

That said, if we decide to examine the question of plant intelligence comparatively, we can take advantage of experimental models and paradigms that have already been utilized to study intelligent behavior in animals. Not that plants and animals are the same thing, but these models can facilitate our comparison of how plants and animals perceive environmental cues. What may emerge from our study of plant and animal behaviors is the realization that they complement each other nicely and, if nothing else, demonstrate once again just how similar all free-living organisms are to one another.

As a comparative psychologist, I study motor cognition in humans and other animal species using the reach-to-grasp movements as an experimental model. It is well known that the processes transforming the perceptual features of objects into suitable motor patterns for grasping is a hallmark of intelligence [14]. When a hand meets an object, the overlapping worlds of sensorimotor and cognitive functions connect [15]. Plants do not have hands, of course. Nonetheless, the oscillatory movements of the tendrils of climbing plants reaching towards a potential support are mesmerizing. There is something about that movement that makes me think of the coordinated hand-reaching movements preparing to grasp an object. Naturally, the tips or tendrils of plants move very slowly in a helical pattern to reach a support (to catch more light) (i.e., circumnutation [16]), while hands/arms move quickly in a more linear fashion. Nevertheless, once these movements become normalized over time, the underlying motor control principles seem quite similar to me.

When Darwin [16] conducted experiments on climbing plants, he reported observing that vines were able to locate supports and to lean towards them; instead, when the support was perceived as excessively thick or smooth, the vines leaned away from it. Although anecdotal, his reports corroborate the theory that climbing plants can modify their circumnutation patterns ‘anticipating’ some features of the targeted support.

With this in mind, we decided to take a closer look at the phenomenon by speeding up the movement of pea plants using time-lapse techniques and applying three-dimensional (3D) kinematical analysis to video recordings. We used classic paradigms to study motor cognition in pea plants. First of all, we set out to see if the plants adjust their circumnutation pattern and the aperture of their tendrils depending on the thickness of the potential supports [17,18,19,20]. We observed that the plants’ kinematic scaling, which was found to depend on the supports’ thickness, resembles hand pre-shaping for reach-to-grasp movements towards an object by a human, a non-human primate, a rat and to some extent even by a bird attempting to grasp with its beak. The plants were also able to process some of the support’s properties even before they made contact with it. Finally, like the animals, and in accordance with a well-known principle (Fitt’s law), they strategically and anticipatorily modulated their movement velocity depending on the task difficulty [21,22]. According to that principle, the amount of time required to perform an action involving a target is a function of the distance to the target divided by the size of the target. Thus, the longer the distance and the smaller the target’s size, the longer it takes [23]. Moreover, the plants corrected their movement in flight, adopting the same strategy animals use for tasks requiring greater precision [24]. When the task involves reaching to grasp a smaller target, slower movements facilitate acquiring more information about the target and any spatial discrepancy there may be between the effector/target, which lead to appropriate adjustments [25]. A far cry from being a simple cause-effect mechanism, the movement of pea plants seems to be a smoothly controlled and executed exercise.

However, “how” an action is performed is not determined exclusively by biomechanical constraints; it also depends on the context within which the action is performed. Some experiments have been carried out to investigate if plants’ reach-to-grasp movements are affected by the presence/absence of a neighbor [26]. Experiments were designed with a single plant in a pot located near a single support for an individual context and two plants in the same pot with a support in the middle for a social context. The results of the experiments showed that there were differences in the kinematics depending on the context. In the presence of a neighbor, the plants modified their behaviors to maximize their long-term gains, including that of grasping a potential support. Overall, these data suggest that plant kinematics are modulated depending upon the context within which their movement towards a potential support occurs as they adjust their behavior in such a way as to improve their chances for survival.

In the face of evidence of ‘intelligent’ behavior on the part of pea plants, it is time we take a judicious look at traditional definitions of intelligence based on arbitrary, outdated, human-centric (mis)conceptions. Of course, all hypotheses need empirical confirmation at both the behavioral and the physiological levels, and species-specific tests need to be carried out using a pluralistic interdisciplinary approach. As knowledge about plant intelligence and cognition expands, the similarities between plant and animal behaviors are becoming increasingly evident. Future research will seek to answer questions regarding both plant sensitivity and responses to stimuli allowing them to make adjustments and/or to optimize growth as well as the features of intelligence that plants and animals share.
